# Using the β-glucosidase catalyzed reaction product glucose to improve the ionic liquid tolerance of β-glucosidases

**DOI:** 10.1186/s13068-016-0484-3

**Published:** 2016-03-22

**Authors:** Shubhasish Goswami, Neha Gupta, Supratim Datta

**Affiliations:** Department of Biological Sciences, Indian Institute of Science Education and Research Kolkata, Mohanpur, 741246 India

**Keywords:** Biomass pretreatment, Ionic liquid, β-Glucosidase, Half-life, Glucose tolerance, Thermostability, Cellulase, Uncompetitive inhibition

## Abstract

**Background:**

Pretreating biomass with ionic liquids (IL) increases enzyme accessibility and cellulose is typically recovered through precipitation with an anti-solvent. An industrially feasible pretreatment and hydrolysis process requires robust cellulases that are stable and active in the presence of either small amounts of ILs co-precipitated with recovered cellulose or for saccharifications in the presence of IL. β-glucosidase (BG) hydrolyzes cellobiose into two molecules of glucose (Glc) and is the last step of biomass hydrolysis. These enzymes are prone not only to product inhibition by glucose but also to inactivation by ILs. With increasing interest in IL-based pretreatment methods, there is increasing focus toward a search for Glc-tolerant and IL-tolerant BG.

**Results:**

We identified a BG belonging to the GH1 family, H0HC94, encoded in Agrobacterium tumefaciens 5A, and cloned and overexpressed the protein in *Escherichia coli.* H0HC94 exhibited high enzymatic activity with β-glycosidic substrates (248 µmol/min/mg on pNPGlc and 262 µmol/min/mg on cellobiose) and tolerant to Glc (apparent *K*_i_ = 686 mM). Further evidence of Glc-based stabilization came from the increase in melting temperature of H0HC94, with increasing Glc concentrations. The half-life of H0HC94 also increased between 2- and 20-fold in the presence of increasing concentrations of Glc. In the presence of 0.9 M of different [C_2_mim]-based ionic liquids, the specific activity of H0HC94 decreased by around 20–30 %. However, the addition of 100 mM glucose to the IL-enzyme mix resulted in a more stable enzyme as evidenced by the slight recovery of H0HC94 melting temperature and up to tenfold increase in half-life. This higher stability came at a cost of 2–10 % decrease in specific activity. The steady-state kinetic analyses for a subset of the ionic liquids tested indicate that the enzyme undergoes uncompetitive inhibition by glucose and ionic liquid, indicating the possibility of binding of the ionic liquid and glucose to the enzyme–substrate complex.

**Conclusions:**

H0HC94 is a Glc-stabilized BG that is also tolerant up to 0.9 M concentrations of different IL’s and indicates the possibilities of using an IL–Glc-based cellulose solvent that displays enzyme-compatibility.

**Electronic supplementary material:**

The online version of this article (doi:10.1186/s13068-016-0484-3) contains supplementary material, which is available to authorized users.

## Background

Lignocellulosic biomass, the biomass derived from cell walls of plants such as trees, shrubs, and grasses, is the most abundant plant material on our planet. It is economical and is probably even more considered to save from lower carbon dioxide emissions compared to fossil fuels. Typical sources include agricultural and forest residues, municipal waste such as organic and paper waste and dedicated biofuel crops. However, while sucrose, extracted from sugar cane or sugar beet, can be directly utilized for fermentation toward biofuel production, the cellulose in lignocellulosic biomass needs to be first depolymerised by cellulases to glucose. Cellobiohydrolase’s, endoglucanase’s, and β-glucosidase’s working synergistically to degrade biomass, comprise the minimum set of enzymes known as cellulases [1, 2]. Endoglucanases (EC 3.2.1.4) randomly cleave the β-1,4 glycosidic linkages of cellulose; cellobiohydrolases (EC 3.2.1.91) attack cellulose chain ends to produce cellobiose (a dimer of glucose linked by a β-1,4 glycosidic bond); and β-glucosidases (EC 3.2.1.21) hydrolyze cellobiose into two molecules of glucose (Glc). β-glucosidase (BG) catalyze the hydrolysis of the β-1,4 linkage between two glucose molecules in a cellulose polymer. The hydrolysis step is generally recognized as the major limiting step in the development of efficient enzyme-based technologies for the conversion of lignocellulosic biomass to sugars due to the accumulation of the reaction product, glucose [3]. Relieving this glucose inhibition is therefore a major challenge.

This enzymatic depolymerisation step requires the cellulose to be accessible to the enzymes, thus necessitating the pretreatment of the biomass, traditionally via dilute acids, strong bases, lime, organosolv etc. [4]. While there are a wide range of physical, chemical, and combined approaches for biomass pretreatment, one method that has recently attracted much attention is the use of ionic liquids [5]. This is because strong solvents represent a risk due to hazardous industrial wastes with negative consequences to air and soil pollution, climate change etc. IL’s are green solvents typically consisting of an organic cation and either an organic or inorganic anion, and hold great promise for biomass pretreatment [6]. Though cost of IL’s have been a frequent criticism against the use of IL’s, the advantages of IL pretreatment have stimulated research in the design of low-cost IL’s and IL recovery [7–9]. The low enzyme activities in aqueous solutions of cellulose-dissolving IL’s are however an important drawback [10]. There are previously published reports on the activity, stability, and action in hydrolysis experiments of mesophilic *Trichoderma reesei* and *Aspergillus niger* cellulase systems as well as thermophilic sources, either with mostly endoglucanases or as cellulase cocktails, and very few BG [10–13]. The metrics for cellulolytic performance in IL include activity measurements, stability (retained cellulolytic activity after incubation in IL), and long-term hydrolysis experiments. Readers are referred to a nice compilation of the effects of IL on cellulases by Wahlström et al. [14]. The strategies toward the search for the ionic liquid-tolerant enzymes started with assaying enzyme activities of known cellulases in the presence of ionic liquids as well as using metagenomic analyses to selecting enzymes tolerant to IL [6, 15–17]. Another approach had been to introduce polyethylene glycol (PEG) chains into the cation of the IL [10, 18]. Jones and Vasudevan immobilized *T. reesei* cellulase by cross-linking the enzyme with glutaraldehyde and measured an enhancement in enzyme activity in the presence of 2 % (v/v) of 1-ethyl-3-methylimidazolium diethyl phosphate [19]. Cellulase immobilization onto sodium alginate, a polymeric support, Amberlite XAD4 coated with hydrophobic IL, and on chitosan has also been reported [20]. We wanted to test the saccharification reaction product Glc, which is also an osmolyte, as part of a strategy to stabilize BG’s in the presence of IL and chose a mesophilic cellulase since thermophilic enzymes have been previously reported to have enhanced IL tolerance [16]. To determine specific activities and effect of IL, we decided to assay in 0.9 M of ionic liquid 1-ethyl-3-methylimidazolium acetate ([C_2_mim][OAc]) and other [C_2_mim]-based IL’s, since these conditions are particularly relevant for the deconstruction of biomass through IL pretreatment [13]. Substituted imidazoles have been shown in the literature to cause inhibition of cellulases and the inhibition amounts are dependent on the substituent types and positions on the imidazole ring [21]. After getting a handle on the effect of [C_2_mim]-based IL’s, we planned to enhance the IL tolerance toward our goal of an enzyme compatible to in situ pretreatment and saccharification.

Therefore, in this study, the putative gene encoding β-glucosidase in the pathogenic bacteria *Agrobacterium tumefaciens* 5A, H0HC94, was cloned and expressed in *Escherichia coli*. The recombinant enzyme was purified, and its biochemical properties, including optimum pH and temperature, thermostability, substrate specificity, and saccharification of cello-oligosaccharides, were investigated. The kinetic data in the absence and in presence of exogenously added Glc were fit to the Michaelis–Menten model by non-linear regression analysis. The enzyme was found to be uncompetitively inhibited by Glc, with an apparent *K*_i,app_ = 686 mM. No transglycosylation activity, in the presence of Glc, could be detected. The *t*_1/2_ of H0HC94 increases with increase in Glc concentrations, indicating stabilization by Glc. Alongside, the melting temperature (T_m_) of the enzyme increases. While the enzyme had a short half-life in the presence of ILs, as expected for mesophilic enzymes based on existing literature, an increased tolerance to ionic liquids in the presence of 100 mM Glc, both in terms of activity and higher T_m_ and half-life, was detected. The IL are predicted to bind to the enzyme–substrate complex and inhibit the enzyme. 1-ethyl-3-methylimidazolium diethyl phosphate ([C_2_C_1_im][C_2_C_2_PO_4_]), 1-ethyl-3-methylimidazolium dimethyl phosphate ([C_2_C_1_im][C_1_C_1_PO_4_]), and 1-ethyl-3-methylimidazolium l-(+)-lactate ([C_2_C_1_im][MeCHOHCO_2_]) were indeed found to uncompetitively inhibit H0HC94. This is one of the first reports of a Glc-stabilized β-glucosidase that is also more stable to increased concentrations of IL in the presence of Glc. The ability of cellulases to remain active in IL-Glc co-solvents sets the stage for developing a continuous process for conversion of biomass to energy [22].

## Results and discussion

### Cloning, expression, and purification of β-glucosidase from *A. tumefaciens*

**Fig. 1:**
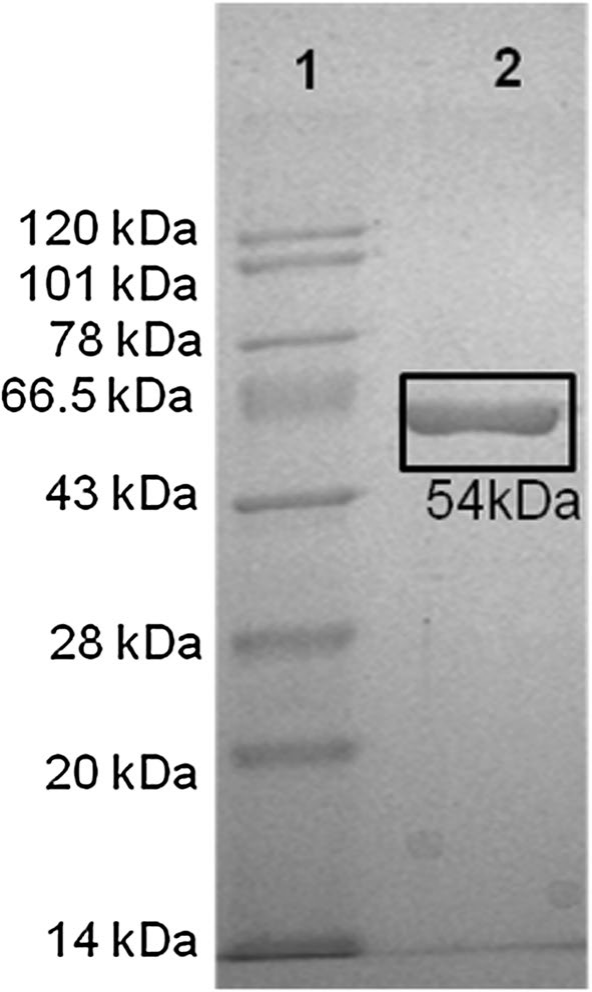
SDS-PAGE of purified H0HC94. *Lane 1* molecular markers; *Lane 2* fraction after affinity chromatography

A gene of 1380 bp encoding a β-glucosidase from *A. tumefaciens*, with the same sequence as that reported in GenBank (EHJ97046.1), was cloned (as described in “Experimental” section) and expressed in *E*. *coli*. The enzyme was purified from crude extract obtained from harvested cells as a soluble protein via His-Trap affinity chromatography. Proteins obtained at each purification step were analyzed by SDS-PAGE, and the finally purified enzyme showed a single band with an apparent molecular mass of 54 kDa (Fig. 1). A molecular mass of 54 kDa agrees well with the predicted size of H0HC94 (52 kDa) plus the His tag and polylinker regions (2 kDa) and verified by MALDI-MS (data not shown). The purification yielded about 50 mg of pure protein per liter of *E. coli* culture, the purity being around 90 %. The purified enzyme preparation had a specific activity of 248 ± 5 U mg of protein^−1^ (1 U = 1 µmol of pNP formed per min per mg of H0HC94). Till date, there are two reported BG’s from *Agrobacterium*, *A. tumefaciens* sp. (P27034) from a GH3 family and *Agrobacterium* sp. (P12614) from a GH1 family [23–25]. *Agrobacterium tumefaciens* 5A (H0HC94), shares a 94 % sequence similarity with *Agrobacterium* sp. (P12614) (Additional file 1: Figure S1). The sequence similarity is however negligible between the *A. tumefaciens* (H0HC94) and *A. tumefaciens* sp. (P27034).

## Biochemical characterization of H0HC94

### Temperature and pH optima

**Fig. 2:**
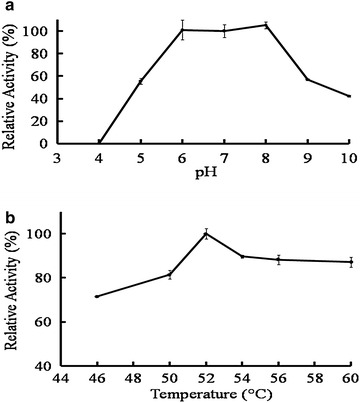
pH and temperature profile of H0HC94. The effect of the wide range of the both temperature and the buffers had been measured using standard β-glucosidase assay. **a** The pH optimum (pH_opt_) was found to be between pH 6 to pH 8. **b** The temperature optimum (T_opt_) was found to be 52 °C. Data are expressed as percent specific activity and represent mean ± SD of three-independent experiments, each performed in duplicate

The temperature dependence and pH dependence of purified H0HC94 are shown in Fig. 2. H0HC94 showed substantial activity at temperatures from 50 to 60 °C and exhibited the highest activity at the optimal temperature of 52 °C. The enzyme shows the highest activity across a wide pH range from 6.0 to 8.0 (Fig. 2), as evident from the maximal activity between these pH ranges, when assayed with 20 mM pNPGlc at 52 °C. At pH 5 and 9, the relative activity of the enzyme was 55 % of the maximal activity, showing the relative stability of the enzyme across an even wider range of pH.

### Thermostability

The thermostability of the enzyme was investigated by measuring the residual activity after incubating H0HC94 at 4, 25, and 52 °C for different time periods. Under the assay conditions used (50 mM phosphate buffer, pH 7.2, 5 min at 52 °C), H0HC94 was highly stable at both 4 and 25 °C with no loss in activity observed after 24 h of incubation at 4 and 25 °C. The enzyme was however quickly inactivated when incubated at 52 °C, with a half-life of 16 min (vide infra). The pH stability was also investigated by measuring the residual activity after 24 h of incubation at 4 °C at pH ranging from 4 to 10. The enzyme was stable at pH 5–9.0 with no appreciable loss of activity at 4 °C. At pH 4.0, measurement of specific activity post overnight incubation produced no measureable activity.

### Substrate specificity and kinetic constants

The hydrolytic activity of H0HC94 toward various substrates was measured to determine substrate specificity. Among the chromogenic substrates, not only pNPGlc but also pNPGal were good substrates of H0HC94 (Table 1). The enzyme showed very little activity with pNPC (4 % with respect to pNPGlc) and zero activity with pNPMal (data not shown). The steady-state kinetic parameters of H0HC94 were measured to determine the Michaelis–Menten kinetic parameters by varying the substrate concentration and the data fit using a non-linear regression method, for pNPGlc and cellobiose (Clbs) under optimal conditions (5 min, pH 7.2, 52 °C). The enzyme had apparent *K*_m_ values of 0.55 and 15.8 mM and *k*_cat_ values of 277 and 233 s^−1^ for hydrolysis of pNPGlc and Clbs, respectively. H0HC94 showed a greater hydrolytic efficiency for pNPGlc than the natural substrate Clbs and pNPGal. The *k*_cat_ ranks among the highest listed for GH1’s and GH3’s [22, 26, 27].

**Table 1:** Comparison of the kinetic parameters of H0HC94 with different BG substrates

Organisms	T_opt_,°C	pH_opt_	Substrates	*K*_m_, mM	*k*_cat,_ s^−1^	*k*_cat_/*K*_m_ (mM^−1^s^−1^)
H0HC94 (*Agrobacterium tumefaciens 5A*)	52	7.2	pNPGlc	3.09 ± 0.40	277.9 ± 4	89.94
			pNPGal	12.93 ± 2.00	214.9 ± 7	16.62
			Clbs	2.94 ± 0.20	233.4 ± 6	79.32

All assays were performed as indicated in “Experimental” section

## High d-Glc tolerance of H0HC94

To understand the effect of Glc on H0HC94 in the presence of increasing concentrations of Glc, the steady-state kinetic parameters were determined in the presence of 100, 200, 600, 400, and 800 mM of Glc and compared to when no Glc was exogenously added. While the activity of H0HC94 decreased with increasing concentrations of d-Glc (Table 2), this decrease was less compared to most enzymes reported in literature [26]. In the presence of 600 mM exogenously added d-Glc, and 0–40 mM pNPGlc as substrate, the *k*_cat_ of pNPGlc was around a half of the *k*_cat_ in the absence of Glc. When the *v* vs. [pNPGlc] was fit to an uncompetitive inhibition Michaelis–Menten model by a non-linear regression fit, the apparent inhibition constant, *K*_i,app,_ was determined to be 686 mM of Glc. This *K*_i,app_ is among the highest and accurately reported (based on the non-linear regression fit), for a bacterial β-glucosidase from a known organism [26, 28].

**Table 2:** The effect of glucose on the steady-state kinetic parameters (*K*_m_, *k*_cat_, *K*_i, app_), melting temperature (T_m_), and half-life (*t*_1/2_) on HOHC94

Glucose (mM)	*K*_m_ (mM)	*k*_cat_ (s^−1^)	*k*_cat_/*K*_m_	*K*_i, app_ (mM)	Δ*T*_m_ (°C)	*t*_1/2_ (min)
0	3.09	277.93	89.94	–	0	16
100	3.55	236.71	66.67	686	2.4 ± 0.19	38
200	2.64	207.79	78.70		3.3 ± 0.06	44
400	1.89	171.98	90.99		6.7 ± 0.21	160
600	1.68	138.33	82.33		7.7 ± 0.16	289
800	1.37	120.31	113.37		8.7 ± 0.26	310

All assays were performed as indicated in “Experimental” section

The standard deviation of *k*_cat_ and *K*_m_ are within 5 % of the original values

The T_m_ of enzyme in the absence of glucose was 53.73 ± 0.2. For Δ*T*_m_, the *T*_m_ in the absence of glucose was subtracted from each data

All half-life measurements were measured in triplicate and repeated at least twice

While most of the BG reported in literature are competitively inhibited by glucose [29–32], a non-competitive type of inhibition has also been reported [33]. In competitive type of inhibition, higher Glc tolerance has been reported to be accompanied by higher *K*_m_ [26]. Here, however, the *K*_m_ decreases with increasing glucose concentrations, indicating an uncompetitive type of inhibition. The glucose is conjectured to bind to a unique site. Yang et al. have proposed the existence of such glucose binding site other than the active site and ascribe glucose tolerance to such a binding site [34]. Previously, Zanoelo et al. have also proposed the existence of a unique site for xylose and glucose to activate the BG from *Scytalidium thermophilum* [35]. Temperature has also been reported to be an important factor in glucose inhibition, with decreasing glucose inhibition at increasing temperatures [36]. However, H0HC94 has an optimum temperature of 52 °C which is similar to many of the other BG from fungus or bacteria from unidentified bacteria with even higher glucose tolerance [36].

### No transglycosylation activity of H0HC94

**Fig. 3:**
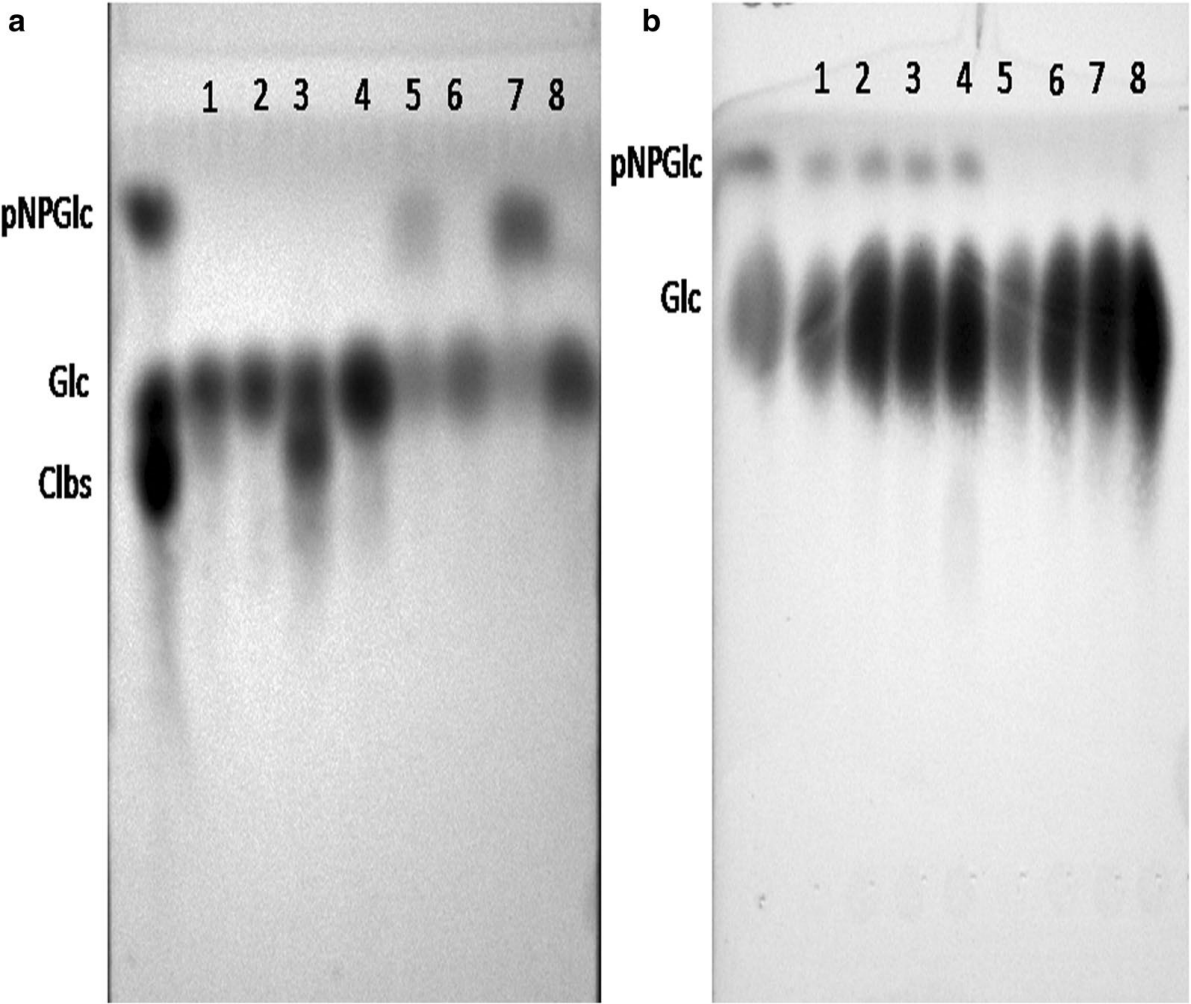
Verifying the presence or absence of a transglycosylation product. The reaction products were analyzed by TLC on activated silica plates by using a *n*-butanol-*n*-propanol-ethanol–water eluent (2:3:3:2). **a** Enzymatic product analysis of substrates cellobiose (Clbs) and pNPGlc at 10 and 20 mM and measured after 5 min and 4 h. *Lane* 1 and 2 represent the products of the 10 mM Clbs reaction after 5 min and 4 h, respectively. *Lanes* 3 and 4 represent the products of the 20 mM Clbs reaction after 5 min and the 4 h, respectively. *Lanes* 5 and 6 represent the products of the 10 mM pNPGlc reaction after 5 min and 4 h, respectively. *Lanes* 7 and 8 represent the products of the 20 mM pNPGlc reaction after 5 min and 4 h, respectively. No transoligosaccharide products band was observed in any one of the reactions indicating absence of the transglycosylation. **b** Similar assays were done as in **a**, but in the presence of Glc. *Lanes* 1–4 indicates glucose concentration ranging from 0.25, 0.5, 0.75, 1 M Glc, respectively, for 5 min. *Lanes* 5–8 indicates glucose concentration ranging from 0.25, 0.5, 0.75, and 1 M Glc, respectively, for 4 h. No transoligosaccharide products band was observed in any of the reaction indicating absence of transglycosylation reaction in the presence of exogenous glucose

To check whether transglycosylation had a role to play in this Glc tolerance, samples were incubated similar to conditions used during the steady-state kinetic assay, both in the absence of Glc as well as in presence of Glc using both pNPGlc and Clbs as substrate (Fig. 3). The reactions products were analyzed by thin layer chromatography (TLC) to verify the transglycosylated product using *n*-butanol: ethanol: *n*-propanol: water (2:3:3:2)-based solvent. Figure 3a shows a comparison across 10 and 20 mM cellobiose and pNPGlc and at 5 min and 4 h. The two time period was chosen to represent the regular activity assay period as well as a much longer period to allow the detection of any products that might be not be detectable during the 5 min assay. While unreacted pNPGlc or Clbs could be seen in Fig. 3a, no transglycosylation product bands with high retention time, toward the bottom of the TLC plate, could be seen. To check the effect of exogenously added Glc on the hydrolysis of 10 and 20 mM pNPGlc, 0.25 to 1 M Glc was added, across both 5 min and 4 h reactions. The slight smears are a result of the Glc overload and again no transglycosylation products were detected. Therefore, it is unlikely that transglycosylation is a factor in Glc tolerance, something that had been reported in literature for other Glc-tolerant BG [37].

### Increase in half-life of H0HC94 in the presence of Glc

The half-life of the enzyme (*t*_1/2_) at 52 °C without any additive was 16 min and the same at 25 °C was 33 h 36 min. This short *t*_1/2_ at *T*_opt_ is a common feature across many mesophilic enzymes and the half-life is usually longer at temperatures lower than the T_opt_ [38]. However when the enzyme was incubated with Glc and then assayed for activity, the half-life of enzyme at 52 °C increases from 16 min to up to 310 min (800 mM Glc) (Table 2), a 19-fold increase in *t*_1/2_. The Glc here is shown to stabilize the enzyme up to 800 mM. While such an increase for a BG has not been previously reported in literature, recently Xu et al. have reported that glucose protects *Hypocrea orientalis* β-glucosidase from heat inactivation [22].

### Melting temperature of H0HC94 increases in the presence of Glc

A differential scanning fluorescence experiment allows the monitoring of protein denaturation upon heating, via fluorescent-based detection [39]. The fluorescent dye-based probe preferentially binds the hydrophobic regions of a protein, which are increasingly exposed during protein denaturation. When measured with a real-time PCR instrument, the change in fluorescence can be monitored to plot a thermal melting curve. The mid-point of the melting curve is the temperature at which 50 % of the protein has denatured and is defined as the melting temperature, T_m_, and is a measure of the protein’s inherent thermal stability. The T_m_ of H0HC94 was found to be 53.73 ± 0.2 °C. The change in T_m_, expressed in terms of ∆T_m_ (°C), is the difference between T_m_ measured by adding varying concentrations of Glc in the absence of exogenously added Glc. The value of ∆T_m_ increased from 0 °C (no Glc) up to 8.7 °C (800 mM Glc) as the Glc concentration was increased from 0 up to 800 mM (Table 2). These ∆T_m_ values point toward the stabilization of this enzyme in presence of Glc.

Thus Glc increases the H0HC94 activity and stability, which is also reflected in the increased half-life of the enzyme. Glc has been known to influence the structure of water, solvate the protein surface, and in turn the strength of hydrophobic interactions [40]. Other than the contribution to the thermodynamic stability of H0HC94, the kinetic stability for this enzyme is probably contributed by the shape and the electrostatic properties of the entrance to the active site of H0HC94, including the glucose-binding subsite inside the active site tunnel [41]. The thermodynamic stability does not always lead to an increase in kinetic competency. The complex interplay between these two factors could result in the increase in H0HC94 Glc tolerance and stabilization but decreased activity.

## Effect of [C_2_C_1_im]-based IL’s on H0HC94

The imidazolium-based IL’s have been extensively studied due to their stability, the ease of synthesis, and effective pretreatment of biomass through effective π-stacking and hydrogen bonding interactions with lignin [42]. The ionic liquid anion plays an important role in determining an ionic liquid’s ability to dissolve cellulose [5]. Ionic liquids previously identified in literature to contain anions that can form strong hydrogen bonds with hydroxyl groups include chloride, carboxylates (acetate, formate, propionate, lactate), dialkyl phosphates, methanesulfonate, and amino acid anions [43, 44]. H0HC94 was assayed in the presence of 1-ethyl-3-methylimidazolium methane sulfonate ([C_2_C_1_im][MeSO_3_]), 1-ethyl-3-methylimidazolium diethyl phosphate ([C_2_C_1_im][C_2_C_2_PO_4_]), 1-ethyl-3-methylimidazolium dimethyl phosphate ([C_2_C_1_im][C_1_C_1_PO_4_]), 1-ethyl-3-methyimidazolium acetate ([C_2_C_1_im][MeCO_2_]), 1-ethyl-3-methylimidazolium l-(+)-lactate ([C_2_C_1_im][MeCHOHCO_2_]), 1-ethyl-3-methylimidazolium chloride ([C_2_C_1_im][Cl]), and compared with the organic solvent dimethyl formamide (DMF). The assays were preformed under optimum pH and temperatures for H0HC94.

**Fig. 4:**
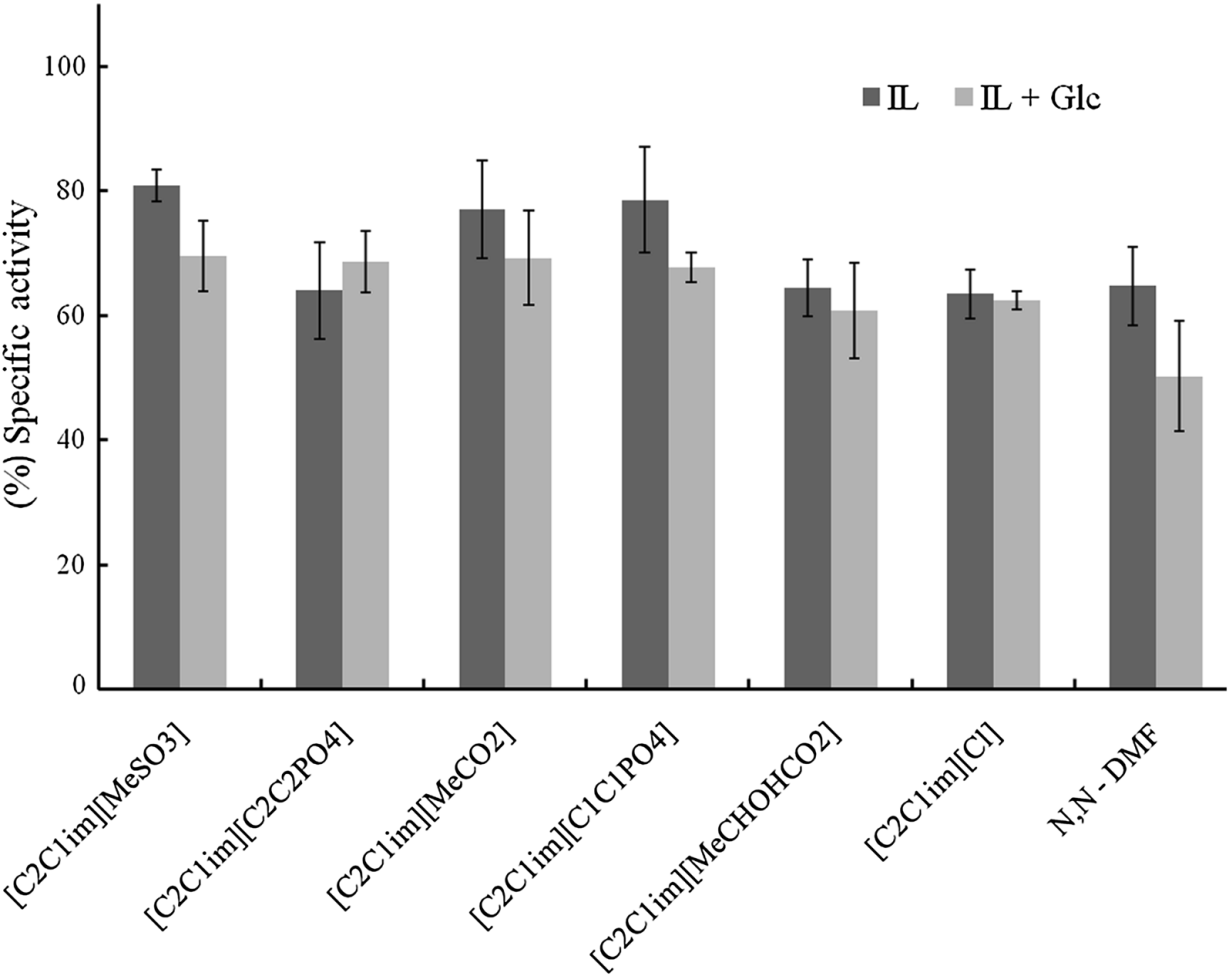
Effect of IL on H0HC94. Comparison of  % specific activity of H0HC94 in 0.9 M of ionic liquids (IL’s) and DMF, both in the absence and presence of 100 mM glucose (Glc). 100 % specific activity ≡ Specific Activity of H0HC94 = 248 U/mg

As is known from previous reports, cellulase from *T. viride*, a mesophilic enzyme, rapidly loses activity with increasing concentration of [C_2_C_1_im][MeCO_2_] and no activity is detectable in the presence of 0.62 M [C_2_C_1_ im][MeCO_2_] [16]. H0HC94 is a similar mesophilic cellulase and we expected a quick decrease in enzyme activity. However, as can be seen in Fig. 4, H0HC94 in the presence of 0.9 M [C_2_C_1_im][MeCO_2_] loses only around 25 % of its specific activity. The highest residual activities were observed with [C_2_C_1_im][MeSO_3_] and [C_2_C_1_im][C_1_C_1_PO_4_], both of which retains 80 % activity in 0.95 M IL. The retention of around 70 % of activity for most of the IL’s tested here (except [C_2_C_1_im][Cl]) might be a consequence of the universal distribution of the negative charge over several atoms that confers minimum changes in enzyme conformation [43, 45].

Since H0HC94 had already been shown to have a high tolerance of glucose (*K*_i,app_ = 686 mM (vide infra), we wanted to test the effect of glucose on H0HC94 incubated with IL. As seen in Fig. 4, the specific activity is either unaffected or slightly decreased in the presence of glucose and IL compared to only IL. While glucose in this case does not have a large effect on the enzyme kinetics, glucose could be inhibiting the enzyme-IL complex. To understand the kinetics of the IL and glucose on the enzyme, the steady-state kinetics was measured in the presence of IL and compared with the measurements in the presence of 0.9 M IL and 100 mM glucose. Three IL’s were selected based on activity and half-life, namely, [C_2_C_1_im][C_1_C_1_PO_4_], [C_2_C_1_im][C_2_C_2_PO_4_], and [C_2_C_1_im][MeCHOHCO_2_]. The results indicated in Table 3, show that all of the three [C_2_C_1_im]-based IL’s uncompetitively inhibit the enzyme, presumably binding to the enzyme though there are no experimental evidence as yet. Jaeger et al. used docking studies and MD simulations to show that catalytic inhibition in presence of ionic liquid is due to the binding of cations to active site pocket of a xylanase, resulting in competitive inhibition [46].

**Table 3:** The effect of IL (0.9 M) on the steady-state kinetic parameters (*V*_max_, *K*_m_, *k*_cat_) of H0HC94 (E) in the absence and the presence of 100 mM Glc

Reaction	*V*_max_ (mM)	*k*_cat_ (s^−1^)	*K*_m_ (mM)	*k*_cat_/*K*_m_ (s^−1^ mM^−1^)
E	0.31	277.93	3.09	89.94
E + [C_2_C_1_im][C_1_C_1_PO_4_]	0.43	188.11	2.17	86.49
E + [C_2_C_1_im][C_1_C_1_PO_4_] + 0.1 M Glc	0.27	116.71	1.50	77.49
E + [C_2_C_1_im][C_2_C_2_PO_4_]	0.38	165.79	4.20	39.43
E + [C_2_C_1_im][C_2_C_2_PO_4_] + 0.1 M Glc	0.25	107.50	2.59	41.60
E + [C_2_C_1_im][MeCHOHCO_2_]	0.33	143.56	13.26	10.83
E + [C_2_C_1_im][MeCHOHCO_2_] + 0.1 M Glc	0.26	113.16	12.31	9.19

The standard deviation of *k*_cat_ and *K*_m_ are within 5 % of the original values

### Melting temperature of H0HC94 increases in the presence of Glc and IL

Since glucose increases the ∆T_m_ of H0HC94 and point toward a Glc-induced stabilization of H0HC94, we wanted to also understand how IL and the combination of IL and Glc affect enzyme stability. As seen in Table 4, while the addition of 100 mM Glc results in an increase of ΔT_m_ of H0HC94 by 1.2 °C, addition 0.9 M of each of the IL’s tested, results in a decrease in ΔT_m_ by 5–10 °C. This was not unexpected since IL’s had been previously reported to destabilize cellulases and in particular mesophilic cellulases compared to enzymes from thermophiles or hyperthermophiles. Interestingly, β-glucosidases in general have been known to be less tolerant to IL’s, though the reasons are not known [47]. The organic solvent DMF decreased the enzyme stability by similar amounts as the tested IL’s. Thus while all of the solvents decreased the stability of the H0HC94, the decrease in the presence of IL was not due to ionic interactions only.

**Table 4:** Melting temperature (*T*_m_) of H0HC94 in 0.9 M IL’s, DMF, and NaCl, both in the absence and presence of 100 mM glucose (Glc)

	T_m_(°C) in 0.9 M IL	∆T_m_	T_m_(°C) in 0.9 M IL + 100 mM Glc	∆T_m_
H0HC94 (E)	53.73 ± 0.40	0.0	–	–
E + Glc	54.93 ± 0.85	1.20	–	–
E + [C_2_C_1_im][MeSO_3_]	46.54 ± 0.68	−7.19	48.37 ± 1.38	−5.36
E + [C_2_C_1_im][C_2_C_2_PO_4_]	44.64 ± 1.90	−9.09	46.73 ± 1.85	−7.00
E + [C_2_C_1_im][MeCO_2_]	44.50_-_ ± 1.62	−9.23	46.58 ± 1.18	−7.15
E + [C_2_C_1_im][C_1_C_1_PO_4_]	48.44 ± 2.50	−5.29	49.24 ± 1.14	−4.49
E + [C_2_C_1_im][MeCHOHCO_2_]	44.86 ± 1.50	−8.87	47.99 ± 1.85	−5.74
E + [C_2_C_1_im][Cl]	44.87 ± 1.13	−8.86	45.30 ± 0.59	−8.43
E + DMF	44.21 ± 0.44	−9.52	45.40 ± 2.21	−8.33

∆T_m_ was calculated by subtracting the zero IL H0HC94 T_m_ (53.73 °C) from the T_m_ of H0HC94 in 0.9 M IL and also from the T_m_ of H0HC94 0.9 M IL and Glc co-solvent. Data are expressed in °C and represent mean ± SD of three independent experiments, each performed in triplicate

We next tested the enzyme under similar conditions in the presence of IL, but in the presence of 100 mM Glc (Table 4). For most of the IL’s tested, the ΔT_m_ decreased by approximately 2 °C less (or resulted in increase of T_m_ by approx. 2 °C) than in the presence of only IL, thus indicating a stabilizing effect by Glc. Only in the case of [C_2_mim][Cl] does Glc not play any role possibly due to high hydrogen bond basicity of the chloride ion that maximizes interaction with the core of the enzyme and hence destabilizing H0HC94 [45, 48].

### Increase in half-life of H0HC94 in the presence of Glc and IL

To verify whether the stability gained in the presence of glucose results in kinetic benefits, we measured the half-life of H0HC94 in the presence of IL (Table 5). We see that in the presence of three of the IL’s tested along with 100 mM Glc, the half-life of H0HC94 increased by around 150–900 %. This relatively large increase in half-life suggests the possibility of using Glc as a co-solvent to stabilize the enzyme in reactions with IL. This would be applicable to both, left-over IL from a pretreatment reaction or the IL during a simultaneous pretreatment and saccharification reaction. Glucose concentrations below the apparent *K*_i_ and generated during the saccharification reaction could be the source of this glucose, in addition to the possibility of an exogenous addition to stabilize the enzyme during the reaction. This strategy in conjunction to engineering better activity and glucose tolerance in BG’s could be the key to a much better and industrially relevant cellulase.

**Table 5:** Comparison of *t*_1/2_ of H0HC94 in the presence of IL (0.9 M) and DMF (1.6 M) with *t*_1/2_ of H0HC94 in the presence of IL (0.9 M) and DMF (1.6 M) and 100 mM Glc

	*t*_1/2_(min)	*t*_1/2_(min) in 100 mM Glc	% Increase of *t*_1/2_ in 100 mM Glc
H0HC94 (E)	16	38	138
E + [C_2_C_1_im][MeSO_3_]	20	200	900
E + [C_2_C_1_im][C_2_C_2_PO_4_]	48	215	348
E + [C_2_C_1_im][MeCO_2_]	40	220	450
E + [C_2_C_1_im][C_1_C_1_PO_4_]	50	280	460
E + [C_2_C_1_im][MeCHOHCO_2_]	40	160	300
E +[C_2_C_1_im][Cl]	6	15	150
E + DMF	7	30	300

The increase of *t*_1/2_ of H0HC9 4 in the presence of Glc is reported as  % increase in the last column

## Conclusions

Our results show that H0HC94 is a β-glucosidase with higher turnover numbers compared to previously characterized BG’s from *Agrobacterium* or to other BG’s from identified bacterial micro-organisms. H0HC94 is stabilized by its reaction product Glc, resulting in increased half-life and T_m_. While BG’s are generally known to be very sensitive to IL, H0HC94 in the presence of Glc and all of the IL’s tested, has an enhanced half-life and shows the possibilities of using an IL-Glc-based cellulose solvent that is enzyme-compatible.

## Experimental

### Chemicals

All chemicals used were reagent grade. Restriction endonucleases, DNA ligase, and DNA polymerase were purchased from NEB (MA, USA). Primers were synthesized by Xcelris (India). All chromogenic substrates and ionic liquids were purchased from Sigma-Aldrich. The active fractions post-purification were pooled and concentrated using 30 kDa cut-off size membranes of Amicon-Ultra-15 (Millipore).

### Bacterial strains, culture conditions, and plasmids

*Agrobacterium tumefaciensLBA 4404* was used as a source of genomic DNA. *A. tumefaciens* cells were grown overnight at 30 °C at 200 rpm in LB medium (10 g/L tryptone, 5 g/L yeast extract, 10 g/L NaCl, pH 7.2). 1.5 ml culture was used to isolate genomic DNA. *E. coli* BL21(DE3) (Stratagene Cloning Systems, La Jolla, CA) was used as the cloning host for the T7 RNA polymerase expression vector pET-21b(+) (Novagen, Madison, USA). It was grown in Luria–Bertani medium supplemented with 100 μg/ml ampicillin.

### Isolation of genomic DNA

Isolation of genomic DNA from *A. tumefaciens* was performed using the protocol of Sambrook et al. [49].

### Primer design, PCR, and cloning

Flanking primers (Fwd 5′ AGGCCCAAGCTTATGACCGATCACAAAGCG-3′ and Rev 5′ TTGCCGCTCGAGCCCCTTCATCACACCGTGG-3′) containing *Xho*I and *Hind*III restriction sites were designed according to the DNA sequence of H0HC94 present on NCBI. Purified genomic DNA from *A. tumefaciens* was used as a template, and the DNA fragments were amplified by PCR using the primers. PCRs were done on a Veriti thermal cycler (Life Technologies, USA). The reactions were carried out with Phusion DNA polymerase (New England Biolabs, Beverly, MA). The annealing temperature was 59 °C, and the extension time was 45 s. PCR products were separated by 0.8 % agarose gel electrophoresis and extracted from the gel using the QIAquick Gel Extraction kit (Qiagen, USA). The extracted DNA fragments were digested with restriction enzymes and ligated into the pET-21b(+) vector linearized with the same enzymes. The ligation product was transformed into DH5α and the construct verified by restriction analysis and DNA sequencing. For expression studies, verified construct was transformed in BL21(DE3) competent cells.

### Protein expression and purification

Escherichia coli BL21(DE3) transformed with the plasmid construct was grown in Luria–Bertani (LB) medium containing ampicillin (100 μg mL^−1^) at 37 °C, induced with 0.5 mM IPTG (at *A*_600 nm_ = 0.6), and then grown for 8 h at 30 °C. H0HC94 with a C-terminal His tag was purified from cells centrifuged from a 100 ml culture and suspended into 10 mM potassium phosphate/150 mM NaCl buffer (pH 7.2) with 10 mM imidazole containing 1 mM PMSF, 1.2 mg mL^−1^ lysozyme, and one tablet of EDTA-free protease arrest (G-Bioscience, USA). After sonication and centrifugation, the supernatant was loaded onto a Ni-nitrilotriacetic acid column (GE Healthcare, Piscataway, NJ) equilibrated with phosphate buffer containing 150 mM NaCl and 10 mM imidazole. The column was washed extensively, and proteins were eluted with a 10–500 mM linear imidazole gradient. The proteins were eluted between 400 and 500 mM imidazole and were pooled and dialyzed against 50 mM phosphate buffer (pH 7.2). The protein concentrations were measured by Bradford Assay and through extinction coefficient calculated using the ExPASy ProtParam tool [50]. Molar absorption coefficient was calculated to be 104280 M^−1^ cm^−1^. The approximate protein yields were around 50 mg/L. The recombinant protein purity was visually assessed using SDS-PAGE.

### Enzyme activity assays

The activity of H0HC94 was assayed for 5 min at 52 °C in 50 mM potassium phosphate buffer (pH 7.2) using substrate containing the non-physiological chromogenic aglycone *p*-nitrophenol (pNP), e.g., pNPGlc, pNPGal, and pNPMal. The reaction mixture was pre-heated at 52 °C for 5 min, before the addition of enzyme as the last ingredient and then assayed for 5 min. The total reaction volume when using the chromogenic substrates was 100 µl and typically contained 0.2 µg of enzyme. To stop the reaction, 100 µl of 0.4 M glycine pH 10.8 maintained with 50 % NaOH, was added to the reaction; the pNP generation was recorded at 405 nm; and the concentration of pNP was determined using a standard graph, prepared under the same conditions. One unit of β-glucosidase activity is expressed as the amount of enzyme required to release 1 μmol of pNP per minute under the above assay conditions. All substrate concentrations and enzyme activity are reported over 100 µl reaction.

The activity of enzyme with cellobiose was determined by measuring the amount of glucose liberated as a product. Glucose oxidase-peroxidase assay (Glucose Oxidase kit, Sigma) was used to measure the glucose in accordance with the manufacturer’s protocol adapted to microplate assay. The reducing sugar concentration in the sample was calculated from its absorbance using the standard curve of d-glucose. In all assays, spontaneous hydrolysis of the substrate was accounted for by assay of blank mixtures, which lacked the enzyme. All measurements were performed in triplicate.

The pH dependence of the H0HC94 was determined by measuring the specific activities of the enzyme on *p*-nitrophenyl-d-glucopyranoside (pNPGlc) in the range of pH of 4.0–10.0 at 52 °C for 5 min. The effect of temperature on enzyme activity for pNPGlc was measured from 46 to 60 °C, while incubating in 50 mM potassium phosphate buffer at pH 7.2 for 5 min.

For hydrolysis reactions in the presence of IL’s, control reactions with pNPGlc but without enzyme were subtracted from each measurement.

### Kinetic analysis of H0HC94

The kinetic parameters of H0HC94 were determined using pNPGlc and cellobiose as the substrates. The reaction conditions and the methods used to detect enzymatic activity are described above. The reaction velocity was determined at eight different substrate concentrations from 0.8 to 20 mM, *K*_m_ for each substrate. The kinetic constants *K*_m_ and *k*_cat_ were calculated by a non-linear regression of the Michaelis–Menten equation using GraphPad PRISM version 5.0 (GraphPad Software, La Jolla, CA).

The steady-state kinetics in the presence of IL, glucose, and both, were measured as reported earlier and kinetic constants were determined by a non-linear regression of the modified Michaelis–Menten equation in the presence of inhibitors, using GraphPad PRISM version 5.0.

### Thermostability and half-life assay

The thermostability of the purified enzyme was investigated by incubating the enzyme solutions in 50 mM potassium phosphate buffer, pH 7.2, at 52 °C, and under different concentrations of glucose and the respective IL’s. At different time intervals, aliquots were taken and centrifuged, and were assayed with 20 mM pNPGlc at 52 °C for 5 min. Activity of enzyme was monitored by measuring the pNP generation at 405 nm in order to determine the half-life. Half-life times were calculated using the equation for a one-phase exponential decay in the prism graphing program.

### Differential scanning fluorimetry

Melt curve study of protein was performed by measuring the fluorescence of the SYPRO Orange dye (Sigma, USA) at *λ*_ex_ 492 nm (FAM) and *λ*_em_ 610 nm (ROX) using the Step One Plus real-time PCR detection System (Applied Biosystems, USA). Protein melt curve was studied from 30 to 95 °C in 1 °C per min increments and data were collected after each degree increase. Typically, 2 μl H0HC94 (final concentration 6 μM) and 5 μl SYPRO Orange (final concentration 10X) were added to MicroAmp^®^ Fast 8-Tube Strip, and 0.1 mL (Applied Biosystems) was added to a final volume of 25 μl with 50 mM phosphate buffer pH 7.2. The protein was screened against glucose (final concentration 0–800 mM), 15 % (v/v) IL’s and glucose with IL’s. Each measurement was made in triplicates and at least repeated thrice. The unfolding of protein was recorded by fluorescence intensity verses temperature plot. Data transformation and analysis were performed using the DSF Analysis protocol as described [39].

### Transglycosylation

To check the presence of transglycosylation products, H0HC94 was incubated with different concentrations of pNPGlc (10 and 20 mM) and Cellobiose (10 and 20 mM) for 5 min and 4 h under standard kinetic assay conditions (vide infra). Similar reactions were performed where enzyme was incubated with 10 and 20 mM pNPGlc with different concentrations of glucose (0.25–1 M) for 5 min and 4 h at 52 °C. Samples were collected and immediately heated at 95 °C for 5 min to inactivate the enzyme.

The reactions were analyzed by thin layer chromatography (TLC) to verify the transglycosylated product. TLC was carried out using TLC silica plates (TLC silica gel 60 F_254_ Merck, Germany). 0.5 µL of the reaction mix was applied to TLC plates with appropriate dilutions and standards. The solvent used to separate the analytes was *n*-butanol: ethanol: *n*-propanol: water (2:3:3:2). Aniline (0.2 g)–diphenylamine (0.2 g) solution in acetone and 2 ml of phosphoric acid (prepared immediately before use) reagent were used for the detection.

## Additional file

10.1186/s13068-016-0484-3. Sequence alignment of H0HC94 against the previously known *Agrobacterium* β-glucosidases. Full-length amino acid sequences were aligned by ClustalW2 (1) followed by alignment in the Espript 3.X programme (2). The identical residues are shown in white with a black background, and conservative changes are shown in gray background with box. The amino acid sequences used were from *Agrobacterium* sp. (Uniprot accession number P12614); *Agrobacterium tumefaciens* 5A (Uniprot accession number H0HC94).

## Abbreviations

BG: β-glucosidase
Clbs: cellobiose
DSF: differential scanning fluorimetry
DMF: dimethyl formamide
[C_2_mim]: 1-ethyl-3-methylimidazolium
([C_2_C_1_im][Cl]): 1-ethyl-3-methylimidazolium chloride
([C_2_C_1_im][MeCO_2_]): 1-ethyl-3-methylimidazolium acetate
([C_2_C_1_im][MeCHOHCO_2_]): 1-ethyl-3-methylimidazolium l-(+)-lactate
([C_2_C_1_im][C_2_C_2_PO_4_]): 1-ethyl-3-methylimidazolium diethyl phosphate
([C_2_C_1_im][MeSO_3_]): 1-ethyl-3-methylimidazolium methane sulfonate
Glc: d-glucose
GH1: glycoside hydrolase family 1
GH3: glycoside hydrolase family 3
IL: ionic liquid
pNP: *p*-nitrophenyl
pNPGlc: *p*-nitrophenyl-d-glucopyranoside
pNPGal: *p*-nitrophenyl-d-galactopyranoside
pNPMal: *p*-nitrophenyl-d-maltopyranoside
TLC: thin layer chromatography
T_m_: melting temperature
*t*_1/2_: half-life
