# Supplementary material for: Using the β-glucosidase catalyzed reaction product glucose to improve the ionic liquid tolerance of β-glucosidases
Source: Biotechnol Biofuels. 2016 Mar 22;9:72. doi: 10.1186/s13068-016-0484-3 (PMC4802596; doi:10.1186/s13068-016-0484-3)
Supplement: Supplementary file 1 — 10.1186/s13068-016-0484-3. Sequence alignment of H0HC94 against the previously known Agrobacterium β-glucosidases. Full-length amino acid sequences were aligned by ClustalW2 (1) followed by alignment in the Espript 3.X programme (2). The identical residues are shown in white with a black background, and conservative changes are shown in gray background with box. The amino acid sequences used were from Agrobacterium sp. (Uniprot accession number P12614); Agrobacterium tumefaciens 5A (Uniprot accession number H0HC94). [file 13068_2016_484_MOESM1_ESM.doc]

**Supplemental material**

Using the β-glucosidase catalyzed reaction product glucose to improve the ionic liquid tolerance of β-glucosidases

Shubhasish Goswami*^a^*, Neha Gupta*^a^* and Supratim Datta*^a*^*

*^a^*Department of Biological Sciences, Indian Institute of Science Education and Research Kolkata, Mohanpur 741246, India, Fax: 91-33-25873020

*Corresponding author: [supratim@iiserkol.ac.in](mailto:supratim@iiserkol.ac.in)

Figure S1. Sequence alignment of H0HC94 against the previously known *Agrobacterium* β-glucosidases. Full-length amino acid sequences were aligned by ClustalW2 (1) followed by alignment in the Espript 3.X programme (2). The identical residues are shown in *white* with a *black* background, and conservative changes are shown in *gray background with box*. The amino acid sequences used were from *Agrobacterium sp.* (Uniprot accession number P12614); Agrobacterium tumefaciens 5A (Uniprot accession number **H0HC94**);


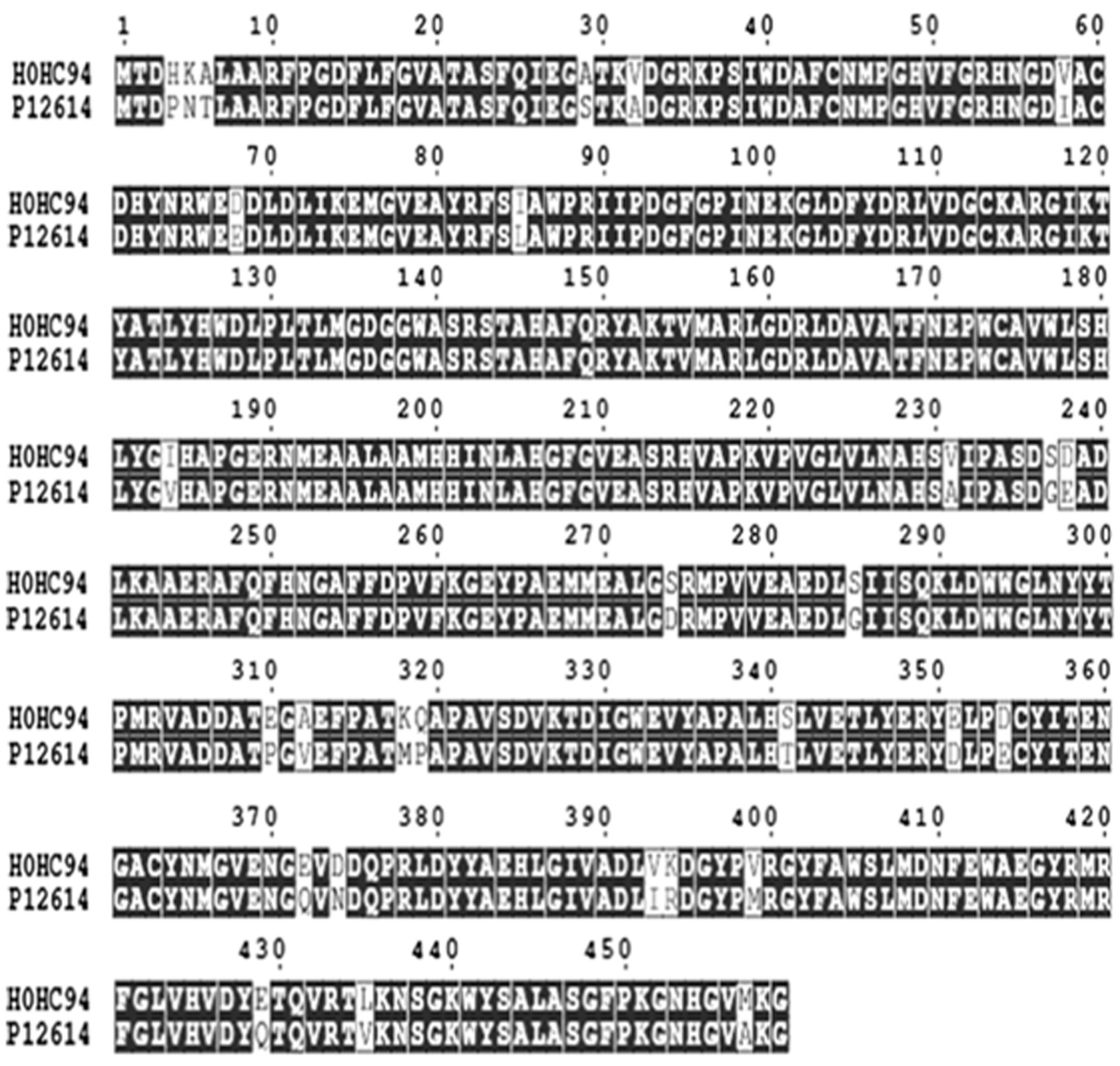


**References**

1. Larkin MA, Blackshields G, Brown NP, Chenna R, McGettigan PA, McWilliam H, Valentin F, Wallace IM, Wilm A, Lopez R, Thompson JD, Gibson TJ, Higgins DG. 
(2007). “Clustal W and Clustal X version 2.0” *Bioinformatics*. 23, 2947-2948

2. Robert, X. and Gouet, P. (2014) "Deciphering key features in protein structures with the new ENDscript server". *Nucl. Acids Res*. **42**(W1), W320-W324
